# Spider mites avoid caterpillar traces to prevent intraguild predation

**DOI:** 10.1038/s41598-023-28861-0

**Published:** 2023-02-01

**Authors:** Shiori Kinto, Toshiharu Akino, Shuichi Yano

**Affiliations:** 1 Laboratory of Ecological Information, Graduate School of Agriculture, Kyoto University, Kyoto, 606-8502 Japan; 2 Applied Entomology Laboratory, Department of Applied Biology, Kyoto Institute of Technology, Kyoto, Kyoto 616-8354 Japan

**Keywords:** Ecology, Ecology

## Abstract

The phytophagous spider mites *Tetranychus kanzawai* and *Tetranychus urticae* can be as small as < 0.5 mm; thus, they are often incidentally consumed along with food plant leaves by voracious lepidopteran larvae (hereafter, ‘caterpillars’). Therefore, the ability to avoid such intraguild predation should confer a selective advantage to mites. We experimentally demonstrated that adult females of both mite species avoided settling on food plant leaves with traces of all tested caterpillar species (*Bombyx mori*, *Papilio xuthus*, *Spodoptera litura* and *Theretra oldenlandiae*). We examined additional interactions using *B. mori* and *T. kanzawai* and found that *B. mori* trace avoidance by *T. kanzawai* lasted for more than 48 h. *Tetranychus kanzawai* also avoided *B. mori* traces on plant stems, along which mites access leaves. Moreover, *T. kanzawai* avoided acetone extracts of *B. mori* traces applied to filter paper, indicating that chemical substances of caterpillar traces are responsible for the avoidance. This study is the first demonstration of a repellent effect of herbivore trace chemicals on heterospecific herbivores. Although spider mites have developed resistance against many synthetic pesticides, these results predict that natural compounds simulating caterpillar traces may repel spider mites from agricultural crops.

## Introduction

Some predators exposed to intraguild predation (IGP) have developed strategies to avoid it^1–4^. By contrast, tiny herbivores are sometimes consumed along with plants by much larger herbivores^5–7^, which can be considered incidental IGP. Therefore, the ability to avoid such incidental IGP should confer a selective advantage to tiny herbivores. However, little is known about IGP avoidance by tiny herbivores except for examples of aphids that immediately drop off a plant in response to the breath of a mammalian herbivore^5^.

The spider mites *Tetranychus kanzawai* Kishida and *Tetranychus urticae* Koch are typically < 0.5 mm in size, and feed on a variety of wild and cultivated plant species^8–10^. These mites construct protective webs on host plant leaves and usually live beneath them^11,12^. Lepidopteran caterpillars such as *Papilio xuthus* L. and *Theretra oldenlandiae* Fabricius that share host plants with these mites^10,13–15^ grow to 30–100 mm, and indiscriminately consume spider mite-infested and uninfested leaves^6^. For example, a final instar *T. oldenlandiae* consumes approximately 20 *Cayratia japonica* (Thunb.) Gagnep. leaflets per day (Kinto, personal observation). Even if some mites can successfully escape caterpillar attack, all eggs and quiescent mites along with webs will be lost^6^. Therefore, any trait that prevents such loss should confer a selective advantage to the mites. Host plant use by *T. kanzawai* and *T. urticae* is ultimately determined by adult females that disperse from their webs and found new webs on uninfested leaves^14–17^ Moreover, adult females of these mites exhibit the ability to detect predator traces on leaves^18–21^. Therefore, we hypothesized that adult female spider mites should avoid caterpillar traces on host plants, which would indicate ongoing caterpillar activity. Here, we provide the first report of spider mite avoidance of caterpillar traces on host plants as well as chemical extracts of these traces.

## Materials and methods

All the materials followed relevant institutional and national guidelines and legislation.

### Mites

We used a *T. kanzawai* population collected from trifoliate orange trees (*Poncirus trifoliata* [L.] Raf.) in 2018 in Kyoto, Japan, and a *T. urticae* population collected from chrysanthemum plants (*Chrysanthemum morifolium* Ramat.) in 1998 in Nara, Japan. These populations were reared on adaxial surfaces of kidney bean (*Phaseolus vulgaris* L.) primary leaves, which were pressed onto water-saturated cotton in Petri dishes (90 mm diameter, 14 mm depth). The water-saturated cotton served as a barrier to prevent mites from escaping. The dishes were maintained at 25 °C, 50% relative humidity, and a 16L:8D photoperiod. All experiments were conducted under these conditions. We only used mated adult females (i.e., the dispersal stage) of *T. kanzawai* or *T. urticae* mites.

### Caterpillars

We used caterpillars of four lepidopteran species: *Bombyx mori* L., *P. Xuthus*, *Spodoptera litura* Fabricius and *T. oldenlandiae*. We collected eggs and larvae of *T. oldenlandiae* from *C. japonica* in 2021 in Kyoto, Japan, and reared them on *C. japonica* leaves until pupation. *Theretra oldenlandiae* shares Vitaceae host plants with *T. kanzawai* and *T. urticae*^8,15^*.* We collected eggs and larvae of *P. xuthus* from *Ptelea trifoliata* in 2021 in Kyoto, Japan, and reared them on *Citrus unshiu* Markov. leaves until pupation. *Papilio. xuthus* and *T. kanzawai* share *P. trifoliata* as a host plant in Kyoto (Kinto, personal observation).

We obtained commercial populations of the *B. mori* Kinshu × Showa strain (Ueda-sanshu Co., Ltd, Nagano, Japan) or the w1-pnd strain. We reared *B. mori* larvae on an artificial diet produced at the Kyoto Institute of Technology. Although *T. kanzawai* use *Morus alba*, a food plant for the *B. mori* strain, the mite and the strain never encounter one another in the wild, because the *B. mori* strain has been domesticated for hundreds of years.

We obtained a sub-cultured population of *S*. *litura* from the Kyoto Institute of Technology. We reared first to fourth instars of *S*. *litura* on an artificial diet (Insecta LFM, Nosan Insect Materials, Kanagawa, Japan), while final instars were fed *P*. *vulgaris* leaves. Because *S*. *litura* feeds on various wild and cultivated plants^22,23^, it may share some host plants with *T*. *kanzawai* and *T*. *urticae*, both of which also feed on many host plant species^8–10^.

We reared caterpillars of *T*. *oldenlandiae*, *P*. *xuthus*, and *S*. *litura* in 900 mL transparent plastic cups and caterpillars of *B*. *mori* in transparent plastic containers (140 × 220 × 35 mm). All caterpillars were maintained under the same laboratory conditions described above.

### Plants

We used several parts of *P*. *vulgaris* plants in the following experiments. This species is a preferred food for both mite species^16,17^ and *S. litura*^24^*,* but the other three caterpillar species do not feed on it (Kinto, personal observation). We thus used *P. vulgaris* rather than shared host plants, because some caterpillars and mites (*T*. *urticae* and *P*. *xuthus*, for example) do not share any host plant.

### Avoidance of caterpillar traces on leaf surfaces by spider mites

**Figure 1:**
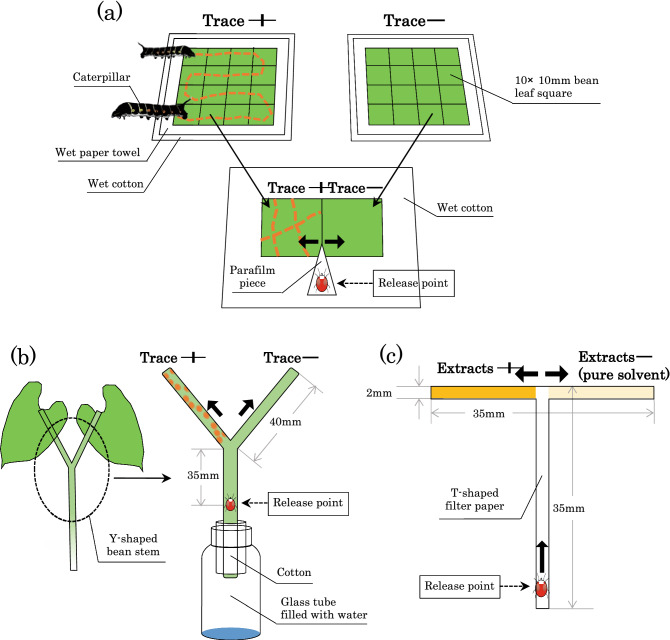
(**a**) Procedure used to observe avoidance of caterpillar traces by spider mites. (**b**) Experimental setup used to observe avoidance of *B*. *mori* traces on plant stems by *T*. *kanzawai*. (**c**) Experimental setup used to observe avoidance of *B*. *mori* trace extracts by *T*. *kanzawai*.

To examine whether spider mites avoid settling on host plant surfaces bearing caterpillar traces, we conducted dual-choice tests using paired adjacent leaf squares with and without caterpillar traces. We did not use whole plants because, in practice, it was difficult to induce caterpillar traces on whole plants. We used two spider mite species (*T*. *kanzawai* and *T*. *urticae*) and four caterpillar species (*T*. *oldenlandiae*,* P*. *xuthus*, *B*. *mori*, and* S*. *litura*). We cut a 10 × 20 mm leaf piece from a fully expanded primary kidney bean leaf and then cut the piece into two equal squares (10 × 10 mm). To introduce caterpillar traces to one square, we arranged them on a separate piece of paper towel on water-saturated cotton. This procedure was necessary because the caterpillars used were larger than individual leaf squares. Then we placed a fourth or final instar caterpillar on the squares and induced the caterpillar to walk across every leaf square three times (Fig. 1a). We carefully removed all caterpillar-produced silk threads from the squares. Within 30 min, we arranged the square (trace +) to touch against the other square (trace −) on water-saturated cotton in a Petri dish. Subsequently, a 2- to 4-day-old mated adult female of *T*. *kanzawai* or *T*. *urticae* was introduced onto a pointed piece of Parafilm in contact with both leaf edges using a fine brush (Fig. 1a). We recorded the leaf square onto which the mite had settled at 2 h after its introduction, as preliminary observations confirmed that all females would settle on a particular leaf within that period. Each female mite and pair of leaf squares were used only once. All tests described below were conducted between 13:00 and 17:00 h, when adult female spider mites actively disperse by walking. There were 14 replicates using traces of *T*. *oldenlandiae*, 48 of *P*. *xuthus*, 20 of *B*. *mori*, and 26 of *S*. *litura* for *T*. *kanzawai*, as well as 18, 32, 16, and 47, respectively, for *T*. *urticae*. Data were subjected to two-tailed binomial tests with the common null hypothesis that a spider mite would settle on the two squares with equal probability (i.e., 0.5).

### Duration of *B. mori* trace avoidance by *T. kanzawai*

To examine whether the effects of caterpillar traces on spider mite avoidance decline over time, we used *T*. *kanzawai* mites and *B*. *mori* caterpillars. We used *B*. *mori* because populations can be easily maintained over many generations. We prepared bean leaf squares with *B*. *mori* traces in the same manner descried above and preserved the traced square on water-saturated cotton for 0 h (n = 30), 24 h (n = 29), 48 h (n = 28), or 72 h (n = 28). Then we arranged the square (trace +) to lie in close proximity to the control square (trace −) that had been preserved for the same periods of time. Then we compared the avoidance response of *T*. *kanzawai* females in the same manner described above.

### Avoidance of *B. mori* traces on plant stems by *T. kanzawai*

To examine whether *T*. *kanzawai* females avoid walking along plant stems bearing caterpillar traces, we used Y-shaped kidney bean stems (Fig. 1b). We cut symmetric bean plants ca. 15 days after sowing from their base and inserted them perpendicularly into a 5 mL glass bottle filled with water and wet cotton. To induce caterpillar traces on one branch of the stem, we allowed a silkworm to crawl from the branching point to the far end of one branch three times for each stem (n = 20). Then we introduced a *T*. *kanzawai* adult female at a release point 35 mm below the branch point (Fig. 1b). We recorded the branch along which the female walked to the far end. Each female mite and each Y-shaped stem were used only once. The numbers of females were compared using binomial tests in the same manner described above.

### Avoidance of *B. mori* trace extracts by *T. kanzawai*

To extract chemical traces of caterpillar, we introduced 10 third instar *B*. *mori* to a glass Petri dish (120 mm diameter, 60 mm depth). After 1 h, we removed all caterpillars and washed the inside bottom of the dish with 1.0 mL acetone. We replicated the procedure twice using different individuals to combine all extracts and to acquire enough extract for the following experiment.

To examine avoidance of *B*. *mori* trace extracts by *T*. *kanzawai* females, we conducted dual-choice experiments using T-shaped pathways of filter paper (35 × 35 mm; width, 2 mm; Fig. 1c). Using disposable micropipettes (Drummond Scientific Co., PA, USA), 1.75 caterpillar equivalents (i.e., 60 µL) of acetone extract were applied to an alternately selected branch (17.5 mm long) of each pathway (i.e., 0.10 caterpillar equivalent/mm), with control acetone applied to the other branch. We applied each solution dropwise at the junction point to minimize mixing. After evaporating the solvent from those pathways, we perpendicularly suspended them (Fig. 1c) and introduced an adult female mite at 2 days post-maturation onto the bottom of each pathway using a fine brush and recorded the branch along which the female first walked to the far end. Each female mite and each T-shaped filter paper were used only once, with 19 replicates. Each female mite made a choice within 10 min. The avoidance response of *T*. *kanzawai* was analysed in the same manner described above.

### Indirect effects of *B. mori* traces on *T. kanzawai* via plants

To determine whether *B. mori* traces on plants indirectly affect the performance of *T. kanzawai* on plants, we introduced 70–80 randomly selected quiescent female deutonymphs of *T. kanzawai* onto kidney bean leaf disks. Immediately after synchronized adult emergence, we introduced the same number of adult males to allow mating; the detailed procedure is described elsewhere^25^. After 24 h, we transferred the females singly onto 10 × 10 mm bean leaf squares with or without *B. mori* traces prepared as described above. Because the number of eggs laid within a certain period is considered the most sensitive performance index of spider mite females^26,27^, any plant-mediated indirect interaction, such as defence induction in response to caterpillar traces, should result in lower egg numbers laid by the test females. We counted the eggs laid on the leaf squares 24 h after their introduction. One female that laid no eggs during the 24 h period (n = 1, trace +) was excluded from the analysis. We obtained 33 and 36 replicates for the trail+ and trail– conditions, respectively. We compared the numbers of eggs laid on leaves with and without *B. mori* traces using a generalized linear model with a Poisson error distribution using the SAS 9.22 software (SAS Institute Inc., Cary, NC, USA).

### Ethics

This article does not contain any studies with human participants or animals.

## Results

### Avoidance of caterpillar traces on leaf surfaces by spider mites

**Figure 2:**
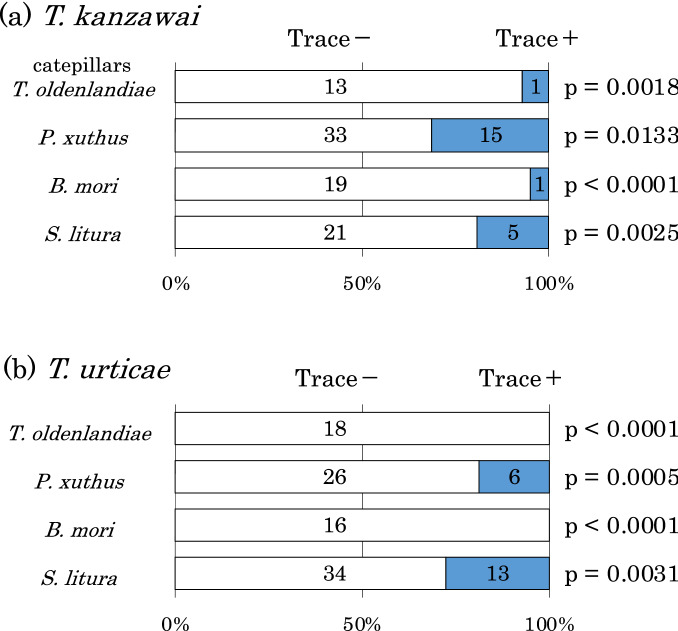
Avoidance of caterpillar traces by (**a**) *T*. *kanzawai* and (**b**) *T*. *urticae*. The numbers of females settled onto each leaf square are provided in each bar.

Significantly fewer *T*. *kanzawai* and *T*. *urticae* females settled onto bean leaf squares with traces of all tested caterpillars (*T*. *oldenlandiae*,* P*. *xuthus*,* B*. *mori*, and* S*. *litura*), indicating that both spider mites avoided traces of these caterpillars (Fig. 2).

### Duration of *B. mori* trace avoidance by *T. kanzawai*

**Figure 3:**
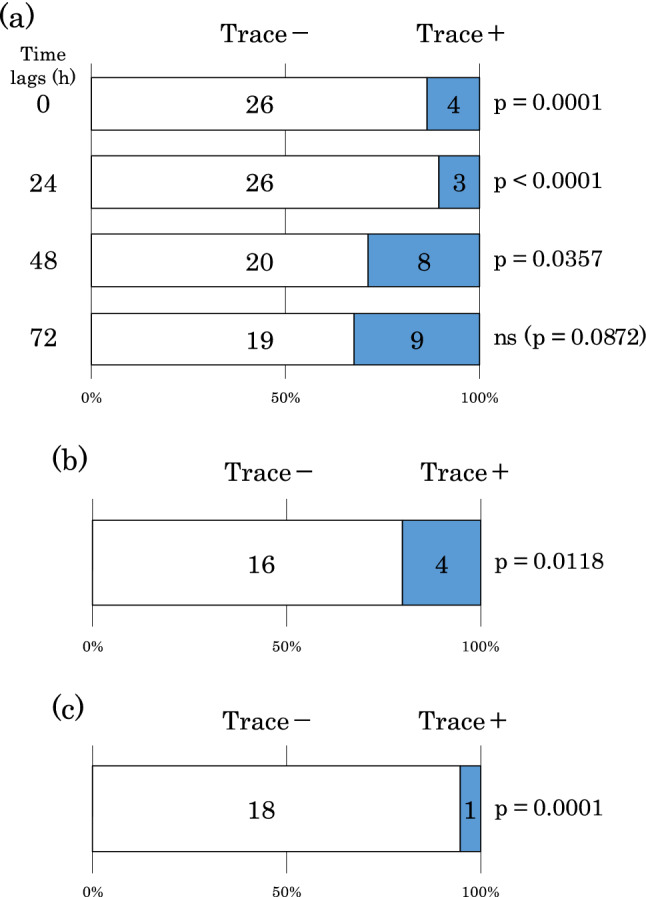
(**a**) Duration of caterpillar trace avoidance by *T*. *kanzawai*. (**b**) Avoidance of *B*. *mori* traces on plant stems by* T*. *kanzawai*. (**c**) Avoidance of *B*. *mori* trace extracts by *T*. *kanzawai*.

*Tetranychus kanzawai* females significantly avoided *B*. *mori* traces that were aged 0 h (26:4, binomial test *P* = 0.0001), 24 h (26:3, binomial test *P* < 0.0001), and 48 h (20:8, binomial test *P* = 0.0357), but not 72 h (19:9, binomial test *P* = 0.0872, Fig. 3a). These results suggest that avoidance lasted for more than 48 h but less than 72 h.

### Avoidance of *B. mori* traces on plant stems by *T. kanzawai*

Significantly fewer *T*. *kanzawai* females walked along branches bearing *B*. *mori* traces (16:4, binomial test *P* = 0.0118, Fig. 3b), indicating that mites avoided walking along caterpillar traces on plant stems.

### Avoidance of *B. mori* trace extracts by *T. kanzawai*

Significantly fewer *T*. *kanzawai* females walked along the branch to which *B*. *mori* trace extracts had been applied (18:1, binomial test *P* = 0.0001, Fig. 3c), indicating that mites avoided caterpillar trace extracts.

### Indirect effects of *B. mori* traces on *T. kanzawai* via plants

We found no significant difference in the number of eggs (mean ± standard error [SE]) laid by *T. kanzawai* on leaf pieces with (9.273 ± 0.449, n = 33) and without (9.778 ± 0.378, n = 36) *B. mori* traces (*P* = 0.4972, GLM), suggesting that any indirect effects of *B. mori* traces on *T. kanzawai* performance on plants were negligible (see Supplementary Data 2).

## Discussion

Both spider mite species avoided traces of all tested caterpillar species. As all silk threads produced by caterpillars were removed before the tests, the caterpillar trace effect seems to be mediated by chemical(s) rather than silk. Although these spider mites avoid predatory mite traces^18–20^, this study is the first to report the avoidance of herbivore traces by spider mites. Spider mites prevent attacks by generalist predators using their protective webs^28–31^. They also prevent attacks by specialist predators that penetrate the webs by dispersing from invaded patches^18,19,32,33^ or by seeking refuge and laying eggs on webs that cannot be easily accessed by the predators^34–38^. However, spider mites would be defenceless against large caterpillars that incidentally, rather than intentionally, consume spider mites along with host plant leaves^6^. For example, *Phytoseiulus persimilis* Athias-Henriot, the most voracious predatory mite species, consumes ca. 20 spider mite eggs per day^39^, whereas final instar larvae of *T*. *oldenlandiae* consume tens to hundreds of spider mite eggs on *C*. *japonica* leaves within as little as 10 min (Kinto, personal observation). Therefore, caterpillars would be more harmful as intraguild predators than as real predators of spider mites under some conditions. As the performance index of *T. kanzawai* females did not differ according to the presence or absence of *B. mori* traces on leaves, the avoidance of *B. mori* traces by *T. kanzawai* females may be promoted by factors other than indirect effects of *B. mori* traces via plants. Thus, we suggest that the avoidance of herbivore traces is driven by the prevention of incidental IGP by voracious caterpillars. Caterpillar trace avoidance would prevent spider mites from encountering caterpillars at the cost of abandoning available plant resources. Such repellence appeared to decline over time, but did last for a few days, suggesting that spider mites can avoid encountering caterpillars that likely remain in the vicinity. We did not use whole plants in the experiments described above. However, the data on avoidance of stem caterpillar traces by mites (Figs. 1b and 3b) are robust. The aboveground parts of many plants consist of leaves and stems, and all leaves are connected by stems, along which mites ambulate to access food leaves. It is thus logical to extrapolate spider mite avoidance of caterpillar traces on such stems to avoidance on entire plants.

Interestingly, spider mites avoided not only traces of caterpillars that they could potentially encounter in the wild, but also those of caterpillars that they never encounter in the wild. For example, *T*. *urticae* and *P*. *xuthus* do not share any host plants, and the domesticated *B*. *mori* strain is not distributed in the wild. Because spider mites are extremely polyphagous and may therefore encounter many caterpillar species on host plants, it is likely that spider mites avoid substances that are commonly contained in the traces of many caterpillar species.

*Tetranychus kanzawai* avoided caterpillar traces on both food plant leaves and plant stems. Because most plant leaves are hierarchically connected by stems, spider mite females that avoid walking along caterpillar traces on a plant stem must abandon all food leaves on that stem, which in turn reduces available food resources for their offspring. Although both *T*. *kanzawai* and *T*. *urticae* exploit a wide range of host plants^9,10^, available food resources for spider mites may be largely limited by the ongoing traces of a great number of caterpillars as well as by many predators. The need to avoid IGP and caterpillar traces may be shared by leaf-mining or sessile herbivores. By examining these possibilities, we may be able to partially answer the long-standing question of why only a portion of available resources are used by herbivores^40,41^.

*Tetranychus kanzawai* also avoided acetone extracts of *B*. *mori* traces, indicating that spider mites avoid chemical components of caterpillar traces. This study provides the first demonstration of a repellent effect of herbivore trace chemicals on heterospecific herbivores. Spider mites use sensory receptors on the forelegs and mouthparts to locate feeding sites^42^. They also detect chemical cues left by predators such as predatory mites and ants^18–21^, suggesting that they may detect such cues left by caterpillars. Although spider mites have developed resistance against many synthetic pesticides^43,44^, it may be possible to manufacture spider mite repellents that simulate caterpillar traces, i.e., natural compounds that are seemingly harmless to humans. Such chemicals, which appear to be effective for a few days, may be valuable for repelling spider mites from agricultural crops. As all experiments were conducted in the laboratory, it is essential to conduct field experiments that confirm the effects of caterpillar traces and to identify the chemicals that mediate avoidance.

## Supplementary Information

Supplementary Information 1.

Supplementary Information 2.

## Data Availability

All data can be found in the Supplementary Data.
